# Worldwide time trends in prevalence of symptoms of rhinoconjunctivitis in children: Global Asthma Network Phase I

**DOI:** 10.1111/pai.13656

**Published:** 2021-09-21

**Authors:** David P. Strachan, Charlotte E. Rutter, Monica Innes Asher, Karen Bissell, Chen‐Yuan Chiang, Asma El Sony, Eamon Ellwood, Philippa Ellwood, Luis García‐Marcos, Guy B. Marks, Eva Morales, Kevin Mortimer, Neil Pearce, Virginia Pérez‐Fernández, Steven Robertson, Richard J. Silverwood, the Global Asthma Network Phase I Study Group, EM Navarrete‐Rodriguez, A López‐Silvarrey Varela, MI Asher, K Bissell, C‐Y Chiang, A El Sony, P Ellwood, L García‐Marcos, K Mortimer, N Pearce, DP Strachan, P Ellwood, E Ellwood, MI Asher, L García‐Marcos, V Perez‐Fernández, E Morales, A Martinez‐Torres, DP Strachan, N Pearce, S Robertson, CE Rutter, RJ Silverwood, J Mallol, M Soto‐Martinez, M Singh, V Singh, S Awasthi, SK Kabra, S Salvi, JV Mérida‐Palacio, SN González‐Díaz, José Eleuterio González, JF Sánchez, A Falade, HJ Zar, A López‐Silvarrey Varela, C González Díaz, L García‐Marcos, M Nour, G Dib, J‐L Huang, S Chinratanapisit, ME Soto‐Quirós, A El Sony, P Vichyanond, P Aguilar, ME Soto‐Quirós, S Barba, M Sabir, L Kumar, V Singh, TU Sukumaran, S Awasthi, SK Sharma, NM Hanumante, R García‐Almaráz, JV Merida‐Palacio, BE Del‐Río‐Navarro, SN González‐Díaz, José Eleuterio González, FJ Linares‐Zapién, MI Asher, JF Sánchez, HJ Zar, C González Díaz, L García‐Marcos, OAA Musa, Y Mohammad, J‐L Huang, P Vichyanond, V Aguirre, M Baeza‐Bacab, A El Sony, S Mohammad, E Cortéz, ME Soto‐Quirós, CH Gratziou, L Kumar, TU Sukumaran, K Chopra, NM Hanumante, MI Asher, BO Onadeko, AD Rubio, L García‐Marcos, K‐H Hsieh, J Mallol, J Shah

**Affiliations:** ^1^ Population Health Research Institute St George’s, University of London London UK; ^2^ Department of Medical Statistics London School of Hygiene & Tropical Medicine London UK; ^3^ Department of Paediatrics: Child and Youth Health Faculty of Medical and Health Sciences University of Auckland Private Bag Auckland New Zealand; ^4^ School of Population Health Faculty of Medical and Health Sciences University of Auckland Private Bag Auckland New Zealand; ^5^ International Union Against Tuberculosis and Lung Disease Paris France; ^6^ Division of Pulmonary Medicine Department of Internal Medicine Wan Fang Hospital Taipei Medical University Taipei Taiwan; ^7^ Division of Pulmonary Medicine Department of Internal Medicine School of Medicine College of Medicine Taipei Medical University Taipei Taiwan; ^8^ Epidemiological Laboratory (Epi‐Lab) for Public Health, Research and Development Khartoum Sudan; ^9^ Paediatric Allergy and Pulmonology Units Virgen de la Arrixaca University Children‘s Hospital University of Murcia and IMIB Bio‐health Research Institute Murcia Spain; ^10^ ARADyAL Allergy Network Edificio Departamental‐Laib Murcia Spain; ^11^ Respiratory & Environmental Epidemiology University of New South Wales Sydney New South Wales Australia; ^12^ Department of Public Health Sciences University of Murcia Murcia Spain; ^13^ IMIB Bio‐health Research Institute Edificio Departamental‐Laib Murcia Spain; ^14^ Liverpool School of Tropical Medicine Liverpool UK; ^15^ Department of Paediatrics University of Murcia Murcia Spain; ^16^ Centre for Longitudinal Studies UCL Social Research Institute University College London London UK

**Keywords:** allergic disease, conjunctivitis, prevalence, rhinitis, time trend

## Abstract

**Background:**

The Global Asthma Network (GAN), by using the International Study of Asthma and Allergies in Childhood (ISAAC) methodology, has updated trends in prevalence of symptoms of childhood allergic diseases, including non‐infective rhinitis and conjunctivitis (‘rhinoconjunctivitis’), which is reported here.

**Methods:**

Prevalence and severity of rhinoconjunctivitis were assessed by questionnaire among schoolchildren in GAN Phase I and ISAAC Phase I and III surveys 15–23 years apart. Absolute rates of change in prevalence were estimated for each centre and modelled by multi‐level linear regression to compare trends by age group, time period and per capita national income.

**Results:**

Twenty‐seven GAN centres in 14 countries surveyed 74,361 13‐ to 14‐year‐olds (‘adolescents’) and 45,434 6‐ to 7‐year‐olds (‘children’), with average response proportions of 90% and 79%, respectively. Many centres showed highly significant (*p *< .001) changes in prevalence of rhinoconjunctivitis in the past year (‘current rhinoconjunctivitis’) compared with ISAAC. The direction and magnitude of centre‐level trends varied significantly (*p *< .001) both within and between countries. Overall, current rhinoconjunctivitis prevalence decreased slightly from ISAAC Phase III to GAN: −1.32% per 10 years, 95% CI [−2.93%, +0.30%] among adolescents; and −0.44% [−1.29%, +0.42%] among children. Together, these differed significantly (*p *< .001) from the upward trend within ISAAC. Among adolescents, centre‐level trends in current rhinoconjunctivitis were highly correlated with those for eczema symptoms (rho = 0.72, *p *< .0001) but not with centre‐level trends in asthma symptoms (rho = 0.15, *p *= .48). Among children, these correlations were positive but not significant.

**Conclusion:**

Symptoms of non‐infective rhinoconjunctivitis among schoolchildren may no longer be on the increase globally, although trends vary substantially within and between countries.

**Key Messages**

Previous studies, mainly in affluent countries, suggest a rising prevalence of hay fever or allergic rhinitis among children and young adults up to the mid‐2000s. This rise was also reported globally by the International Study of Asthma and Allergies in Childhood (ISAAC), based on questionnaires enquiring about non‐infective rhinoconjunctivitis. More recent trends are uncertain, but repetition of ISAAC surveys in 27 centres, including many in low‐ or middle‐income countries, as part of the Global Asthma Network (GAN), permits an updated assessment using standardized methodology. Overall, rhinoconjunctivitis prevalence decreased slightly over 15 years from ISAAC to GAN, among both adolescents (aged 13–14 years) and children (aged 6–7 years). However, the trends observed varied substantially and significantly both within and between countries, limiting the internal and external generalizability of conclusions. Nevertheless, GAN’s global perspective suggests that the prevalence of symptoms of non‐infective rhinoconjunctivitis may no longer be increasing among children, as it was previously. However, due to the heterogeneity of trends observed, local investigation is important to guide local decision‐making.

## 1 INTRODUCTION

Non‐infective rhinitis and conjunctivitis (‘rhinoconjunctivitis’) are common manifestations of allergic disease among children, and their prevalence varied substantially around the world during the 1990s, as documented by the International Study of Asthma and Allergies in Childhood (ISAAC) Phase I.^1^ Approximately seven years later, a comparison of ISAAC Phase III with ISAAC Phase I assessed time trends in annual period prevalence of rhinoconjunctivitis symptoms among almost half a million children from 106 centres in 56 countries.^2^ Although no consistent global pattern emerged, the average prevalence of rhinoconjunctivitis symptoms increased among both 6‐ to 7‐year‐olds and 13‐ to 14‐year‐olds. Greater increases were evident in centres from low‐ and middle‐income countries, but prevalence decreased in many centres with the highest rates in ISAAC Phase I, suggesting that rhinoconjunctivitis symptoms may have peaked in those generally more affluent countries.^2^

In this paper, we extend those earlier ISAAC time trend comparisons to include more recent surveys using identical methodology, which were conducted by the Global Asthma Network^3^ in 27 centres that had previously participated in ISAAC. This offers the opportunity to assess time trends over a longer period in both higher and lower income countries. We sought to evaluate whether the prevalence of symptoms of rhinoconjunctivitis among children has continued to rise, or has plateaued, or indeed started to decline, during the first two decades of the 21^st^ century. We also compared this trend with that for symptoms of asthma (wheeze) and eczema (flexural itchy rash).

## 2 METHODS

The Global Asthma Network (GAN) was established in 2012 as a successor to ISAAC, in collaboration with the International Union Against Tuberculosis and Lung Disease. GAN Phase I, adapting the ISAAC approach and methods, not only focuses upon global surveillance of prevalence and severity of asthma symptoms, but has also included ISAAC questionnaires on symptoms of rhinoconjunctivitis and eczema.

Elsewhere, we have published the rationale and study design for GAN Phase I,^3^, ^4^ the scope of completed fieldwork and its geographical overlap with ISAAC^5^ and the results for time trends in prevalence of asthma symptoms, among GAN Phase I centres that previously participated in ISAAC.^6^

GAN Phase I surveys followed the standardized and validated ISAAC methodology,^7^, ^8^, ^9^, ^10^, ^11^ and a specified protocol.^3^ Cluster sampling was employed, selecting from a geographically defined sampling frame (the ‘study centre’) at least 10 schools at random (or all schools if <10), from which all children of the relevant age (or class or grade) were surveyed. All centres studied 13‐ to 14‐year‐olds (‘adolescents’), who self‐completed written questionnaires at school. Additional inclusion of 6‐ to 7‐year‐olds (‘children’) was optional, and their questionnaires were completed at home by their parents. Sample sizes of at least 1000 and preferably 3000 were sought for each age group.

The symptom definitions used for comparisons in this paper were identical to those used in previous ISAAC rhinitis–related publications^1^, ^2^:

1. ‘rhinitis ever’: a positive answer to the question ‘*Have you [has your child] ever had a problem with sneezing or a runny or blocked nose, when you [he or she] DID NOT have a cold or the “flu?”’*
2. ‘current rhinitis’: a positive answer to ‘*In the past 12 months, have you [has your child] had a problem with sneezing or a runny or blocked nose, when you [he or she] DID NOT have a cold or the “flu?”’*
3. ‘current rhinoconjunctivitis’: ‘current rhinitis’ plus a positive answer to ‘*In the past 12 months, has this nose problem been accompanied by itchy‐watery eyes?’*
4. ‘severe rhinoconjunctivitis’: ‘current rhinoconjunctivitis’ plus an answer of ‘a lot’ to ‘*In the past 12 months, how much did this nose problem interfere with your [child's] daily activities – not at all / a little / a moderate amount / a lot’*.
5. ‘hay fever ever’: a positive answer to the question *‘Have you [has this child] ever had hay fever?’*

Country income category was obtained from the World Bank 2001 data set with countries categorized into low‐, lower‐middle–, upper‐middle– and high‐income countries.^12^

Statistical analysis used Stata version 15.^13^ We derived estimates of the absolute ten‐yearly rate of change in prevalence of rhinitis ever, current rhinitis, current rhinoconjunctivitis, severe rhinoconjunctivitis and hay fever ever for each centre. The standard error (SE) of this change was calculated, allowing for school‐level clustering. Random‐effects meta‐analysis investigated heterogeneity of centre‐level trends within and between countries and age groups.

Additional meta‐analyses compared trend estimates from the ‘earlier period’ (ISAAC Phase I to ISAAC Phase III) and the ‘later period’ (ISAAC Phase III to GAN Phase I) for the subgroup of centres that had participated in all three surveys.

Mixed‐effects linear regression models were used to compare prevalence trends from ISAAC Phase III to GAN Phase I with those from ISAAC Phase I to Phase III (including non‐GAN centres) as previously published.^2^ These models were fitted for each of the five symptom definitions separately. We included country‐ and centre‐level random intercepts to model within‐centre absolute changes in percentage point prevalence per 10‐year interval. Data from both age groups were combined to improve model efficiency but we included age group, region and country income group as confounders and tested for these as effect modifiers.

The relationships between observed centre‐level time trends in rhinoconjunctivitis, asthma and eczema symptoms were assessed by rank correlation. For comparison between trends in the three allergic diseases, we used the sentinel symptoms highlighted in previous ISAAC publications of time trends^14^ and risk factors^15^: ‘current rhinoconjunctivitis’ (for rhinitis symptoms), wheeze in the past year (for asthma symptoms) and itchy rash in the past year with flexural involvement (for eczema symptoms).

## 3 RESULTS

### 3.1 Prevalence results and trends within GAN Phase I centres

GAN survey data, locally checked and centrally collated by January 2021, were available for 119,795 GAN participants from 27 centres in 14 countries that had previously participated in ISAAC Phase I and/or Phase III. These included 74,361 adolescents in 27 centres (13 participating in both ISAAC Phases, 13 in Phase III only and one (Athens) in Phase I only) and 45,434 children in 19 centres (9 participating in both ISAAC Phases, 9 in Phase III only and one (Chandigarh) in Phase I only). Details are shown in Supplementary Tables S1 and S2. On average, GAN fieldwork (March 2015 to February 2020) took place 15.4 years after ISAAC Phase III (April 2001 to October 2003) and 22.7 years after ISAAC Phase I (March 1993 to October 1995). Details of dates of collection and response rates have been published elsewhere.^5^

**FIGURE 1:**
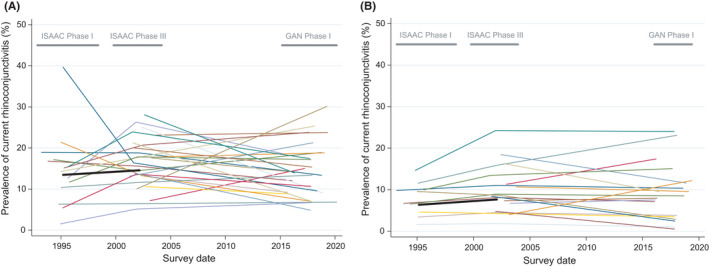
Absolute changes over time in prevalence of current rhinoconjunctivitis (RC) symptoms by mean survey date for 13‐ to 14‐year‐olds (a: left graph) and 6‐ to 7‐year‐olds (b: right graph). Footnote for both subfigures 1a,b: Each coloured thin line represents one GAN Phase I centre. The thick black line shows the average absolute change from ISAAC Phase I to Phase III for those centres that did not participate in GAN Phase I. The span of the years of data collection for ISAAC Phase I, ISAAC Phase III and GAN Phase I is shown

Figure 1 shows the trends in prevalence of current rhinoconjunctivitis for each of the 27 GAN‐ISAAC centres, and (superimposed in black) the average trend in prevalence for ISAAC centres participating in both Phases I and III, but not in GAN. Earlier prevalence data for the non‐GAN centres have been published previously.^2^

Within‐centre trends in current rhinoconjunctivitis varied widely and significantly (*p *< .001) both within and between countries (Tables 1 and 2, Supplementary Figures S1 and S2). On average (pooled random‐effects estimates), current rhinoconjunctivitis prevalence decreased slightly but non‐significantly from ISAAC Phase III to GAN: −1.32% per 10 years, 95% CI [−2.93%, +0.30%] among adolescents; and −0.44% [−1.29%, +0.42%] among children.

**TABLE 1:**
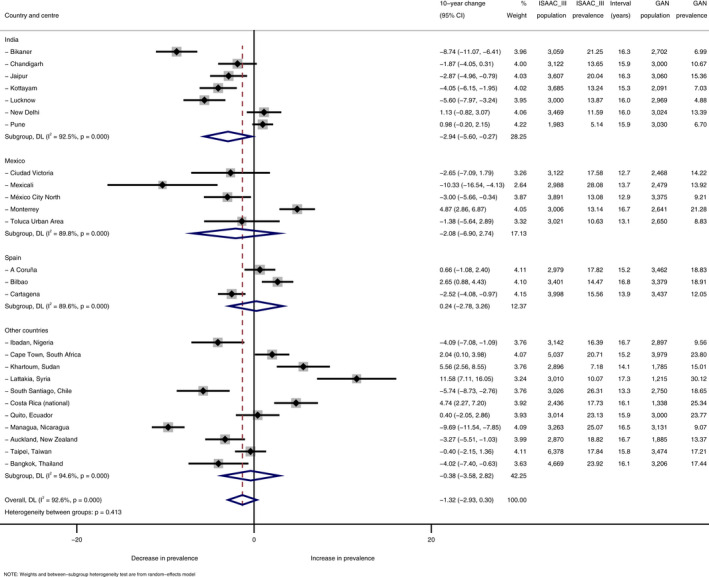
Prevalence trends for current rhinoconjunctivitis from ISAAC Phase III to GAN Phase I among the 13‐ to 14‐year‐old age group, by country and centre

Note

Results expressed as absolute percentage change per 10 years.

**TABLE 2:**
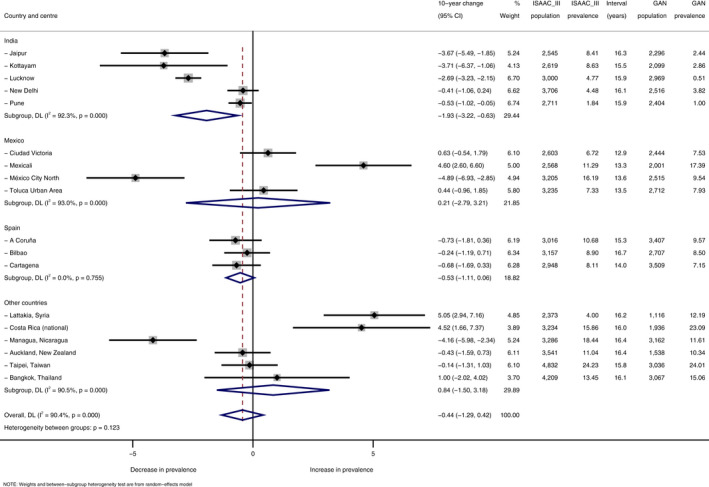
Prevalence trends for current rhinoconjunctivitis from ISAAC Phase III to GAN Phase I among the 6‐ to 7‐year‐old age group, by country and centre

Note

Results expressed as absolute percentage change per 10 years.

Many centre‐specific changes in rhinoconjunctivitis prevalence differed from zero at conventional levels of statistical significance. Substantial and statistically significant diversity was also seen for other common outcomes (rhinitis ever, current rhinitis and hay fever). Even severe rhinoconjunctivitis, with much lower prevalence, changed significantly in several centres in both age groups (Supplementary Tables S1 and S2).

### 3.2 Comparison of within‐centre trends across symptoms, age groups and diseases

Among adolescents, centre‐specific trends in current rhinoconjunctivitis from ISAAC Phase III to GAN correlated very closely with those for rhinitis ever and current rhinitis (both rho = 0.90, *p *< .0001, *N* = 26 centres) and to a moderate but significant degree with trends in severe rhinoconjunctivitis (rho = 0.64, *p *= .0005) and lifetime hay fever (rho = 0.54, *p *= .005). Among children, the corresponding correlations of trends in rhinoconjunctivitis with trends in rhinitis ever, current rhinitis and hay fever were significant but of intermediate strength (rho = 0.5–0.7, *p *< .01, *N* = 18 centres), whereas trends in severe rhinoconjunctivitis were only weakly correlated with those in current rhinoconjunctivitis (rho = 0.27, *p *= .28) (Supplementary Figures S3 and S4).

From ISAAC Phase III to GAN, there was no substantial or significant rank correlation between trends in current rhinoconjunctivitis and the average prevalence of this outcome among adolescents (rho = 0.07, *p *= .73, *N* = 26) nor among children (rho = 0.27, *p *= .27, *N* = 18) (Supplementary Figure S3). When current rhinoconjunctivitis trends were compared between the two age groups, the correlation was weak and non‐significant (rho = 0.38, *p *= .11, *N* = 18).

**FIGURE 2:**
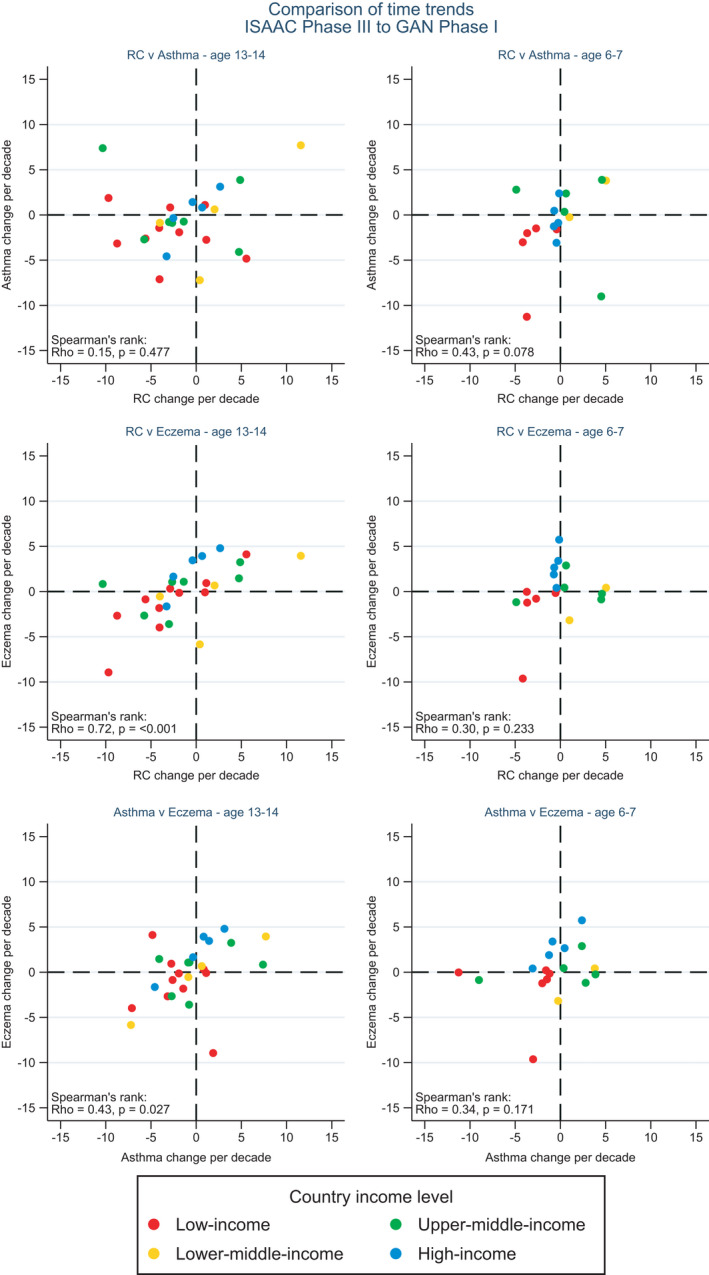
Correlation of centre‐level time trends (absolute percentage change per decade) in prevalence of symptoms of current rhinoconjunctivitis (RC), asthma and eczema from ISAAC Phase III to GAN Phase I, for 13‐ to 14‐year‐olds (left column) and 6 to 7‐year‐olds (right column), countries grouped by GNI per capita

Figure 2 compares within‐centre trends in current rhinoconjunctivitis symptoms with the corresponding trends in symptoms of asthma (wheeze) and eczema (flexural itchy rash), by age group, from ISAAC Phase III to GAN. Although all correlations were positive, only two were statistically significant, both in the adolescent age group (based on 26 centres): rhinoconjunctivitis v eczema (rho = 0.72, *p *< .001) and asthma v eczema (rho = 0.43, *p *= .027). There was only a weak rank correlation between trends in asthma symptoms and current rhinoconjunctivitis among adolescents (rho = 0.15, *p *= .48), and none of the cross‐disease correlations in the younger age group were significant. The correlation between rhinoconjunctivitis trends and eczema trends among adolescents was evident within each of four groups of countries defined by GNI.

### 3.3 Comparison of time trends by period in centres with data at three time points

When the analysis was restricted to centres participating in all three surveys (13 contributing results for adolescents and 9 contributing results for children), the rate of change in prevalence of current rhinoconjunctivitis (pooled across age groups) was significantly (*p *< .001) lower after ISAAC Phase III than before. The inversion in slope (from positive to negative) was similar in both age groups (Table 3). This is consistent with the pattern shown for current rhinoconjunctivitis in Table 4 below.

**TABLE 3:**
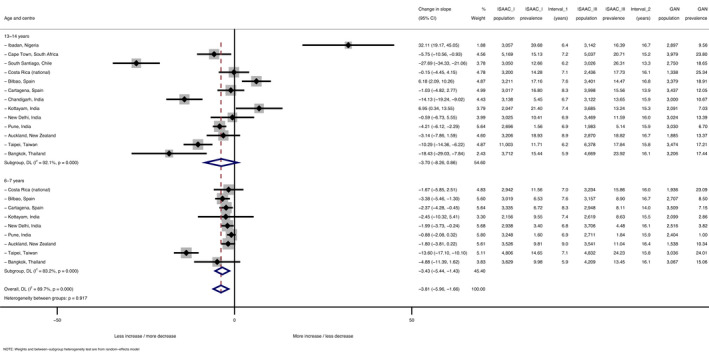
Differences in rate of change of prevalence of current rhinoconjunctivitis, comparing trends after ISAAC Phase III with those before ISAAC Phase III among centres with data at three time points, by age group and centre

Note

Results expressed as absolute percentage change per 10 years.

**TABLE 4:** Modelled estimates of trends in each rhinitis‐related outcome, expressed as absolute percentage change over 10 years

Strata	Absolute percentage change over 10 years (95% CL) by outcome:					Number of surveys
	Rhinitis (no cold or ‘flu) ever	Rhinitis (no cold or ‘flu) past year	Rhinoconjunct‐ivitis past year	Severe rhinoconj‐unctivitis past year	Hay fever ever	
ISAAC Phases I to III, Age 6–7^a^	3.82 (0.30, 7.35)	4.07 (1.31, 6.82)	**2.32 (0.59, 4.04)**	0.12 (−0.08, 0.32)	4.81 (1.84, 7.77)	132
ISAAC Phases I to III, Age 13–14^a^	2.29 (−0.47, 5.06)	3.03 (0.87, 5.19)	**1.28 (−0.08, 2.63)**	0.18 (0.03, 0.34)	4.81 (2.48, 7.14)	214
						
ISAAC Phase III to GAN, Age 6–7^a^	−0.91 (−4.25, 2.42)	−2.31 (−5.27, 0.64)	**−0.41 (−2.34, 1.52)**	−0.04 (−0.25, 0.17)	2.17 (−1.61, 5.95)	36
ISAAC Phase III to GAN, Age 13–14^a^	0.17 (−2.59, 2.93)	−0.53 (−2.97, 1.92)	**−1.15 (−2.75, 0.45)**	−0.14 (−0.32, 0.03)	−0.14 (−3.27, 2.99)	52
					
ISAAC I to III, Low‐income countries^b^	1.65 (−3.99, 7.28)	1.53 (−2.86, 5.91)	**−0.03 (−2.82, 2.76)**	−0.04 (−0.35, 0.28)	8.32 (3.51, 13.12)	52
ISAAC I to III, Lower‐middle‐income^b^	13.96 (7.19, 20.74)	12.04 (6.77, 17.31)	**4.90 (1.54, 8.25)**	0.93 (0.55, 1.31)	2.56 (−3.21, 8.34)	46
ISAAC I to III, Upper‐middle‐income^b^	3.59 (−0.87, 8.06)	5.36 (1.89, 8.83)	**3.20 (0.99, 5.41)**	0.30 (0.05, 0.55)	7.23 (3.43, 11.03)	84
ISAAC I to III, High‐income countries^b^	0.84 (−2.09, 3.76)	1.50 (−0.78, 3.78)	**0.87 (−0.58, 2.32)**	0.01 (−0.15, 0.17)	3.24 (0.75, 5.74)	164
						
ISAAC III to GAN, Low‐income countries^b^	−2.34 (−5.59, 0.90)	−4.10 (−6.91, −1.30)	**−2.94 (−4.76, −1.12)**	−0.34 (−0.54, −0.13)	−3.73 (−7.21, −0.24)	32
ISAAC III to GAN, Lower‐middle‐income^b^	5.35 (0.12, 10.57)	4.17 (−0.35, 8.69)	**2.75 (−0.19, 5.68)**	0.02 (−0.31, 0.35)	−0.02 (−5.64, 5.59)	12
ISAAC III to GAN, Upper‐middle‐income^b^	−1.70 (−5.96, 2.56)	−2.10 (−5.78, 1.58)	**−0.09 (−2.48, 2.30)**	0.06 (−0.21, 0.33)	7.33 (2.75, 11.90)	24
ISAAC III to GAN, High‐income countries^b^	0.95 (−3.22, 5.12)	0.83 (−2.78, 4.43)	**−0.42 (−2.76, 1.92)**	0.06 (−0.21, 0.32)	2.55 (−1.93, 7.02)	20

Note

Results from mixed models with random intercepts for country and centre, by age group and country‐level income group, separately for two time periods.

Estimates for the sentinel symptom “current rhinoconjunctivitis” are shown in bold.

^a^ Adjusted for income group.

^b^ Adjusted for age group.

### 3.4 Modelling of time trends combining GAN and ISAAC data

Multi‐level modelling compared trends in 26 GAN and ISAAC centres (the ‘later period’) with results from 110 ISAAC centres participating in both Phases I and III (the ‘earlier period’). Within each of these two periods, a single centre could contribute data for one or both age groups surveyed at two time points.

Modelling of the combined results for current rhinoconjunctivitis found no significant difference between the age groups (interaction *p *= .28), nor was there effect modification by grouped WHO region (*p *= .31). However, there was significant heterogeneity across country‐level income group (interaction, *p *< .001) and evidence of non‐linearity of the trend across the time period (*p *= .02 for quadratic term).

When earlier and later periods were considered separately (Table 4), the increases for each symptom were greater in the earlier period in each age group, and none of the age‐specific trends from ISAAC Phase III to GAN were significant. The upward trend in current rhinoconjunctivitis in the earlier period was more pronounced and statistically significant in lower‐middle– and upper‐middle–income countries, as previously reported,^2^ and this pattern was similar for other symptoms. During the later period, only lower‐middle–income countries sustained an increase in symptom prevalence from ISAAC Phase III to GAN although this was statistically significant only for rhinitis ever, not for current rhinoconjunctivitis. In contrast, the lifetime prevalence of hay fever increased significantly among upper‐middle–income countries, despite little change in prevalence of the other outcomes (Table 4).

## 4 DISCUSSION

This is the most comprehensive analysis hitherto of time trends in symptoms related to allergic rhinitis among schoolchildren, across diverse study centres around the world using a standardized methodology. We followed ISAAC conventions by focusing on non‐infective rhinitis symptoms accompanied by itchy‐watery eyes, a symptom combination closely related to allergic sensitization, particularly to seasonal allergens, among adults^16^, ^17^ and children^18^, ^19^ in Europe. Even in high‐income countries, atopy appears less relevant to rhinitis without conjunctivitis, and in less affluent settings, the symptom associations with allergic sensitization are much weaker.^19^ Therefore, a global perspective on trends in these symptoms requires cautious interpretation.

Studies in Nordic countries suggest a marked increase in prevalence of allergic rhinitis among children^20^ and older teenagers^21^, ^22^ from the 1980s to mid‐2000s. Elsewhere in Europe, serial prevalence studies of children show a mixed picture: in Switzerland,^23^ the Netherlands^24^ and Poland,^25^ prevalence of rhinoconjunctivitis reached a plateau after the millennium, whereas it continued to increase in Greece.^26^ Outside Europe, the prevalence of doctor‐diagnosed allergic rhinitis among children increased progressively in Turkey from 1994 to 2014,^27^ while a series of 15 large studies of Japanese schoolchildren from 1975 to 2006 showed a continuing increase in the prevalence of seasonal rhinitis and associated itchy eyes.^28^

Our study provides further insight into these long‐term trends in centres mostly outside Europe. Although Brazilian ISAAC centres did not contribute to GAN Phase I, the investigators repeated their 2003 ISAAC fieldwork in 2012 among nine Brazilian centres, which provides time trend data comparable to ours, but over a shorter time period.^29^ A rising prevalence of rhinitis and rhinoconjunctivitis was reported.

Strengths of our study include sample sizes, typically around 3000 per age group, which were large enough to estimate within‐centre trends with adequate precision, allowing for the cluster sampling design. With wide geographical coverage and diverse levels of affluence, we can comment on the patterns of trends internationally, but our most striking observation was of heterogeneity of trends within countries with multiple centres (India, Mexico, Spain), as well as between countries. This limits the extent to which results can be generalized and reduces the statistical power for contrasts such as those between richer and poorer countries.

Despite the smaller number of GAN centres compared with ISAAC and the incomplete overlap between these two lists, sufficient GAN centres had participated in both ISAAC Phases to allow a 3‐point within‐centre analysis. This clearly demonstrates a slowing or reversal of the rate of increase in prevalence of rhinoconjunctivitis previously seen within ISAAC.^2^ This conclusion is robust to inclusion or exclusion of Ibadan, which was a notable outlier in the ISAAC Phase I prevalence data.^1^ Furthermore, it is consistent with the broader comparison of trends in the earlier and later periods, using all available centres irrespective of overlap (Table 4).

Our analysis focused on current rhinoconjunctivitis, but the conclusions generally apply to other rhinitis‐related symptoms, whereas the patterns for trends in lifetime prevalence of hay fever were somewhat different. Hay fever is a label for seasonal allergic rhinitis and/or conjunctivitis in temperate climates but is a less familiar concept in subtropical and tropical regions, where many of our centres are located.

A potential limitation is our reliance upon symptoms reported by adolescents themselves or by parents on behalf of the younger children. No objective tests for allergic sensitization were carried out, nor are any planned. However, the close correlation between within‐centre trends in rhinoconjunctivitis and eczema symptoms (flexural itchy rash) in the adolescent group suggests a common underlying influence. This could be non‐causal (related, for instance, to local awareness or reporting of the two conditions, or to ecological confounding at the centre level) or due to common causal mechanisms. Interestingly, the correlation between rhinoconjunctivitis trends and trends in itchy flexural rash is not limited to the higher income countries. Given the weaker association between atopy and rhinoconjunctivitis symptoms outside of high‐income settings,^19^ it is important that non‐allergic linking mechanisms are sought. The correlations between diseases shown in Figure 2 extend our previous comparisons of trends^30^ and risk factors^15^ for these three related diseases.

## 5 CONCLUSION

The trends we observed varied substantially and significantly both within and between countries, limiting the internal and external generalizability of conclusions. Local investigation is therefore important for understanding local trends and their implications for healthcare decision‐making. Nevertheless, our wide international coverage, including many centres in low‐ or middle‐income countries, provides a global perspective, which suggests that the prevalence of symptoms of non‐infective rhinoconjunctivitis may no longer be increasing among children, as it was previously.

## CONFLICT OF INTEREST

The authors declare that they have no conflict of interest.

## AUTHOR CONTRIBUTIONS

**David P Strachan:** Conceptualization (equal); Formal analysis (lead); Methodology (equal); Supervision (equal); Validation (equal); Writing‐original draft (lead). **Charlotte E Rutter:** Data curation (equal); Formal analysis (supporting); Methodology (equal); Validation (equal); Visualization (equal); Writing‐original draft (supporting). **Innes Asher:** Conceptualization (equal); Funding acquisition (equal); Investigation (equal); Methodology (equal); Project administration (lead); Resources (equal); Writing‐review & editing (equal). **Karen Bissell:** Conceptualization (equal); Writing‐review & editing (equal). **Chen‐Yuan Chiang:** Conceptualization (equal); Methodology (equal); Writing‐review & editing (equal). **Asma El Sony:** Conceptualization (equal); Writing‐review & editing (equal). **Eamon Ellwood:** Data curation (equal); Project administration (supporting); Visualization (equal); Writing‐review & editing (equal). **Philippa Ellwood:** Conceptualization (equal); Data curation (equal); Methodology (equal); Project administration (supporting); Validation (equal); Visualization (equal); Writing‐review & editing (equal). **Luis Garcia‐Marcos:** Conceptualization (equal); Data curation (equal); Methodology (equal); Supervision (equal); Writing‐review & editing (equal). **Guy Marks:** Conceptualization (equal). **Eva Morales:** Data curation (equal); Writing‐review & editing (equal). **Kevin Mortimer:** Writing‐review & editing (equal). **Neil Pearce:** Conceptualization (equal); Formal analysis (supporting); Methodology (equal); Supervision (equal); Validation (equal); Writing‐review & editing (equal). **Virginia Perez‐Fernandez:** Data curation (equal); Validation (equal); Writing‐review & editing (equal). **Steven Robertson:** Data curation (equal). **Richard Silverwood:** Data curation (equal); Methodology (equal); Supervision (equal); Writing‐original draft (supporting). **Global Asthma Network Phase I Study Group:** Investigation (equal); Writing‐review & editing (equal).

### PEER REVIEW

The peer review history for this article is available at https://publons.com/publon/10.1111/pai.13656.

## Supporting information

Supplementary Material

Appendix

## Data Availability

ISAAC data are already deposited for wider use at the UK Data Archive: http://discover.ukdataservice.ac.uk/catalogue?sn=8131 (https://doi.org/10.5255/UKDA‐SN‐8131‐1). The GAN Phase I data, including de‐identified individual participant data, will be made available on the Global Asthma Network website http://www.globalasthmanetwork.org/ within 12 months of all GAN Phase I analyses being published. Access will require a formal request, a written proposal and a signed data access agreement.

## 1 GLOBAL ASTHMA NETWORK PHASE I STUDY GROUP

(Author affiliations are listed in the Supplementary Information.)

**Global Asthma Network Steering Group:** MI Asher, K Bissell, C‐Y Chiang, A El Sony, P Ellwood, L García‐Marcos, GB Marks, E Morales, K Mortimer, N Pearce, DP Strachan.

**Global Asthma Network International Data Centres: GAN Global Centre:** P Ellwood, E Ellwood, MI Asher; **Murcia, Spain:** L García‐Marcos, V Perez‐Fernández, E Morales, A Martinez‐Torres; **London, United Kingdom:** N Pearce, S Robertson, CE Rutter, RJ Silverwood, DP Strachan.

**Global Asthma Network Principal Investigators (* National Co‐ordinators):** J Mallol (South Santiago), M Soto‐Martinez (Costa Rica), A Cabrera Aguilar (Quito), K Douros (Athens), M Sabir (Bikaner), M Singh (Chandigarh), V Singh* (Jaipur), TU Sukumaran (Kottayam), S Awasthi (Lucknow), SK Kabra (New Delhi), S Salvi (Pune), R García‐Almaráz (Ciudad Victoria), JV Mérida‐Palacio (Mexicali), BE Del Río Navarro* (Mexico City North), SN González‐Díaz (Monterrey), EM Navarrete‐Rodriguez (Toluca urban), MI Asher (Auckland), JF Sánchez (Managua), A Falade (Ibadan), HJ Zar (Cape Town) A López‐Silvarrey Varela (A Coruña), C González Díaz (Bilbao), L García‐Marcos* (Cartagena), M Nour (Khartoum), G Dib (Lattakia 13‐14), Y Mohammad* (Lattakia 6‐7), J‐L Huang (Taipei), S Chinratanapisit (Bangkok).

**Global Asthma Network National Coordinators not named above:** ME Soto‐Quirós (Costa Rica), A El Sony (Sudan), P Vichyanond (Thailand).

**ISAAC Phase Three Principal Investigators (* National Co‐ordinators):** P Aguilar (South Santiago), ME Soto‐Quirós* (Costa Rica), S Barba* (Quito), M Sabir (Bikaner), L Kumar (Chandigarh), V Singh (Jaipur), TU Sukumaran (Kottayam), S Awasthi (Lucknow), SK Sharma (New Delhi), NM Hanumante (Pune), R García‐Almaráz (Ciudad Victoria), JV Merida‐Palacio (Mexicali Valley), BE Del‐Río‐Navarro (Ciudad de México), SN González‐Díaz (Monterrey), FJ Linares‐Zapién (Toluca urban), MI Asher* (Auckland), JF Sánchez (Managua), BO Onadeko (Ibadan), HJ Zar* (Cape Town), A López‐Silvarrey Varela (A Coruña), C González Díaz (Bilbao), L García‐Marcos* (Cartagena), OAA Musa (Khartoum), Y Mohammad (Lattakia), J‐L Huang* (Taipei), P Vichyanond* (Bangkok).

**ISAAC Phase Three National Coordinators not named above:** V Aguirre (Chile), M Baeza‐Bacab (Mexico), A El Sony (Sudan), S Mohammad (Syrian Arab Republic).

**ISAAC Phase One Principal Investigators (* National Co‐ordinators):** E Cortéz (South Santiago), ME Soto‐Quirós* (Costa Rica), CH Gratziou* (Athens), L Kumar (Chandigarh), TU Sukumaran (Kottayam), K Chopra (New Delhi), NM Hanumante (Pune), MI Asher* (Auckland), BO Onadeko, (Ibadan), H Nelson (Cape Town), AD Rubio (Bilbao), L García‐Marcos* (Cartagena), K‐H Hsieh^†^ (Taipei), P Vichyanond* (Bangkok).

**ISAAC Phase 1 National Coordinators not named above:** J Mallol (Chile), J Shah (India).

^†^Deceased
