# Supplementary material for: Worldwide time trends in prevalence of symptoms of rhinoconjunctivitis in children: Global Asthma Network Phase I
Source: Pediatr Allergy Immunol. 2021 Sep 21;33(1):e13656. doi: 10.1111/pai.13656 (PMC9012331; doi:10.1111/pai.13656)
Supplement: Supplementary file 2 — Appendix [file PAI-33-e13656-s002.docx]

**Global Asthma Network Phase I Study Group:**

**Global Asthma Network Steering Group:** MI Asher, Department of Paediatrics: Child and Youth Health, Faculty of Medical and Health Sciences, University of Auckland, Private Bag 92019, Auckland, New Zealand; K Bissell, School of Population Health, Faculty of Medical and Health Sciences, University of Auckland, Auckland, New Zealand; C-Y Chiang, International Union Against Tuberculosis and Lung Disease, Paris, France; and Division of Pulmonary Medicine, Department of Internal Medicine, Wan Fang Hospital, Taipei Medical University; and Division of Pulmonary Medicine, Department of Internal Medicine, School of Medicine, College of Medicine, Taipei Medical University, 111 Hsin-Long Road, Section 3, Taipei, 116, Taiwan; A El Sony, Epidemiological Laboratory for Public Health and Research, Khartoum 3 Block3-Building 11, Khartoum, Sudan; P Ellwood, Department of Paediatrics: Child and Youth Health, Faculty of Medical and Health Sciences, Private Bag 92019, University of Auckland, Auckland, New Zealand; L García-Marcos, Pediatric Allergy and Pulmonology Units, Virgen de la Arrixaca University Children‘s Hospital, University of Murcia and IMIB Bioresearch Institute, Murcia; and ARADyAL Allergy Network, Edificio Departamental-Laib, Avenida Buenavista s/n, 30120 El Palmar, 30394 Murcia Spain; GB Marks, Respiratory & Environmental Epidemiology, University of New South Wales, Goulburn St, Sydney 2085, Sydney, Australia; K Mortimer, Liverpool School of Tropical Medicine, Pembroke Place, Liverpool L3 5QA, United Kingdom; N Pearce, Department of Medical Statistics, London School of Hygiene & Tropical Medicine, Keppel Street, London WC1E 7HT, United Kingdom; DP Strachan, Population Health Research Institute, St George’s, University of London, Cranmer Terrace, London SW17 0RE, United Kingdom.

**Global Asthma Network International Data Centres: GAN Global Centre:** P Ellwood, E Ellwood, MI Asher, Department of Paediatrics: Child and Youth Health, Faculty of Medical and Health Sciences, Private Bag 92019, University of Auckland, Auckland, New Zealand; **Murcia, Spain:** L García-Marcos, Pediatric Allergy and Pulmonology Units, Virgen de la Arrixaca University Children‘s Hospital, University of Murcia and IMIB Bioresearch Institute, Murcia; and ARADyAL Allergy Network, Edificio Departamental-Laib, Murcia, Spain; V Perez-Fernández, Department of Paediatrics, University of Murcia; and IMIB Bio-health Research Institute, Murcia, Edificio Departamental-Laib, Avenida Buenavista s/n, 30120 El Palmar, 30394 Murcia Spain; E Morales, Department of Public Health Sciences, University of Murcia, and IMIB Bio-health Research Institute, Murcia, Edificio Departamental-Laib, Avenida Buenavista s/n, 30120 El Palmar, 30394 Murcia, Spain; A Martinez-Torres, Paediatric Allergy and Pulmonology Units and Nurse Research Group, Virgen de la Arrixaca University Children‘s Hospital, University of Murcia and IMIB Bio-health Research Institute, Murcia, Edificio Departamental-Laib, Avenida Buenavista s/n, 30120 El Palmar, 30394 Murcia, Spain; **London, United Kingdom:** DP Strachan, Population Health Research Institute, St George’s, University of London, Cranmer Terrace, London SW17 0RE, United Kingdom; N Pearce, S Robertson, CE Rutter, Department of Medical Statistics, London School of Hygiene & Tropical Medicine, Keppel Street, London WC1E 7HT, United Kingdom; RJ Silverwood, Department of Medical Statistics, London School of Hygiene & Tropical Medicine, Keppel Street, London WC1E 7HT, United Kingdom and Centre for Longitudinal Studies, UCL Social Research Institute, University College London, 20 Bedford Way, London WC1H 0AL, United Kingdom.

**Global Asthma Network Principal Investigators:** Chile: J Mallol, University of Santiago de Chile (USACH), Santiago, (South Santiago); Costa Rica: M Soto-Martinez, University of Costa Rica (Costa Rica); Ecuador: A Cabrera Aguilar Respira Clinic, Planta Baja, Código (Quito); Greece: K Douros, National and Kapodistrian University of Athens (Athens); India: M Sabir, Kothari Medical & Research Institute (Bikaner); M Singh, Postgraduate Institute of Medical Education and Research (Chandigarh); V Singh*, Asthma Bhawan (Jaipur); TU Sukumaran, Pushpagiri Institute Of Medical Sciences And Research, Thiruvalla (Kottayam); S Awasthi, King George's Medical University (Lucknow); SK Kabra, All India Institute of Medical Sciences (New Delhi); S Salvi, Chest Research Foundation (Pune); Mexico: R García-Almaráz, Hospital Infantil de Tamaulipas (Ciudad Victoria); JV Mérida-Palacio, Centro de Investigacion de Enfermedades Alergicas y Respiratorias (Mexicali); BE Del Río Navarro*, Service of Allergy and Clinical immunology, Hospital Infantil de México (Mexico City North); SN González-Díaz, Centro Regional de Alergia e Immunología Clínica, Hospital Universitario ‘Dr. José Eleuterio González’, Universidad Autónoma de Nuevo León (Monterrey); EM Navarrete-Rodriguez, Hospital Infantil de Mexico Federico Gomez (Toluca urban); New Zealand: MI Asher, Department of Paediatrics: Child and Youth Health, Faculty of Medical and Health Sciences, University of Auckland (Auckland); Nicaragua: JF Sánchez, Hospital Infantil Manuel de Jesús Rivera (Managua); Nigeria: A Falade, University of Ibadan and University College Hospital (Ibadan); South Africa: HJ Zar, SA MRC Unit on Child & Adolescent Health (Cape Town); Spain: A López-Silvarrey Varela, Fundacion Maria Jose Jove (A Coruña); C González Díaz, Department of Paediatrics, Universidad del País Vasco UPV /EHU, Bilbao, Spain (Bilbao); L García-Marcos*, Pediatric Allergy and Pulmonology Units, Virgen de la Arrixaca University Children‘s Hospital, University of Murcia and IMIB Bioresearch Institute, Murcia; and ARADyAL Allergy Network, Edificio Departamental-Laib, Murcia, Spain (Cartagena); Sudan: M Nour, Epidemiological Laboratory (Epi-Lab) for Public Health, Research and Development, Khartoum, Sudan (Khartoum); Syrian Arab Republic: G Dib, Lattakia University (Lattakia 13-14); Y Mohammad*, National Center for research and training for chronic respiratory disease and comorbidities (Lattakia 6-7); Taiwan: J-L Huang, Department of Pediatrics, Chang Gung Memorial Hospital, New Taipei Municipal TuCheng Hospital, and Chang Gung University (Taipei); Thailand: S Chinratanapisit, Department of Pediatrics, Bhumibol Adulyadej Hospital, Royal Thai Air Force, Bangkok, Thailand (Bangkok).

* National Coordinators

**Global Asthma Network National Coordinators not named above**

Costa Rica: ME Soto-Quirós, University of Costa Rica, Costa Rica; Sudan: A El Sony, Epidemiological Laboratory for Public Health and Research, Khartoum 3 Block3-Building 11, Khartoum, Sudan; Thailand: P Vichyanond, Mahidol University, Bangkok, Thailand.

**ISAAC Phase Three Principal Investigators:** Chile: P Aguilar, Hospital CRS El Pino, San Bernardo, Santiago, Chile (South Santiago); Costa Rica: ME Soto-Quirós*, University of Costa Rica (Costa Rica); Ecuador: S Barba* AXXIS-Medical Centre SEAICA (Quito); India: M Sabir, Kothari Medical & Research Institute (Bikaner); L Kumar, Department of Pediatrics (Chandigarh); V Singh, Asthma Bhawan (Jaipur); TU Sukumaran, PIMS Thiruvalla (Kottayam); S Awasthi, King George's Medical University (Lucknow); SK Sharma, All India Institute of Medical Sciences (New Delhi [7]); NM Hanumante, Bharati Vidyapeeth Medical College (Pune); Mexico: R García-Almaráz, Hospital Infantil de Tamaulipas (Ciudad Victoria); JV Merida-Palacio, Centro de Investigacion de Enfermedades Alergicas y Respiratorias (Mexicali Valley); BE Del-Río-Navarro, Service of Allergy and Clinical immunology, Hospital Infantil de México (Ciudad de México [1]); SN González-Díaz, Centro Regional de Alergia e Immunología Clínica, Hospital Universitario ‘Dr. José Eleuterio González’, Universidad Autónoma de Nuevo León (Monterrey); FJ Linares-Zapién, Centro De Enfermedades Alergicas Y Asma de Toluca (Toluca); New Zealand: MI Asher*, Department of Paediatrics: Child and Youth Health, University of Auckland (Auckland); Nicaragua: JF Sánchez*, Hospital Infantil Manuel de Jesús Rivera (Managua); Nigeria: BO Onadeko, (Ibadan); South Africa: HJ Zar*, University of Cape Town (Cape Town); Spain: A López-Silvarrey Varela, Fundacion Maria Jose Jove (A Coruña); C González Díaz, Departament de Paediatrics, Universidad del País Vasco UPV /EHU, Bilbao, Spain (Bilbao); L García-Marcos*, Pediatric Allergy and Pulmonology Units, Virgen de la Arrixaca University Children‘s Hospital, University of Murcia and IMIB Bioresearch Institute, Murcia; and ARADyAL Allergy Network, Edificio Departamental-Laib, Murcia, Spain (Cartagena); Sudan: OAA Musa, Faculty of Medicine, National Ribat University, Khartoum, Sudan (Khartoum); Syrian Arab Republic: Y Mohammad, National Center for Research and Training in Chronic Respiratory Diseases—Tishreen University (Lattakia); Taiwan: J-L Huang*, Department of Pediatrics, Chang Gung Memorial Hospital, New Taipei Municipal TuCheng Hospital, and Chang Gung University (Taipei); Thailand: P Vichyanond*, Mahidol University (Bangkok).

* National Coordinators

**ISAAC Phase Three National Coordinators not named above:** Chile: V. Aguirre, University of Santiago de Chile (USACH), Santiago, Chile; Mexico: M Baeza-Bacab, University Autónoma de Yucatán, Yucatán; Sudan: A El Sony Epidemiological Laboratory for Public Health and Research, Khartoum; Syrian Arab Republic: S Mohammad, Tishreen University, Lattakia.

**ISAAC Phase One Principal Investigators:** Chile: E Cortéz, Universidad de Santiago de Chile (USACH), Santiago, Chile (South Santiago); Costa Rica: ME Soto-Quirós*, University of Costa Rica (Costa Rica); Greece: CH Gratziou*, National Kapodistrian University of Athens (Athens); India: L Kumar, Department of Pediatrics (Chandigarh); TU Sukumaran, PIMS Thiruvalla (Kottayam); K Chopra, Maulana Azad Medical College (New Delhi [7]); NM Hanumante, Bharati Vidyapeeth Medical College (Pune); New Zealand: MI Asher*, Department of Paediatrics: Child and Youth Health, University of Auckland (Auckland); Nigeria: BO Onadeko, (Ibadan); South Africa: H Nelson, Horsett Hospital (Cape Town); Spain: AD Rubio, Urgencias de Pediatria. Pabellon Makua, Bilbao, Spain (Bilbao); L García-Marcos*, Pediatric Allergy and Pulmonology Units, Virgen de la Arrixaca University Children‘s Hospital, University of Murcia and IMIB Bioresearch Institute, Murcia; and ARADyAL Allergy Network, Edificio Departamental-Laib, Murcia Spain (Cartagena); Taiwan: K-H Hsieh **^ⴕ^**, Chang Gung Children's Hospital (Taipei); Thailand: P Vichyanond*, Mahidol University (Bangkok).

* National Coordinators

ⴕ Deceased

**ISAAC Phase 1 National Coordinators not named above:** Chile: J Mallol, University of Santiago de Chile (USACH), Santiago, Chile; India: J Shah, Jaslok Hospital & Research Centre, Mumbai.
